# Genome-wide association studies using an adaptive two-stage analysis for a case-control design

**DOI:** 10.1186/1753-6561-1-s1-s147

**Published:** 2007-12-18

**Authors:** Kijoung Song, Qing Lu, Xiwu Lin, Dawn Waterworth, Robert C Elston

**Affiliations:** 1 GlaxoSmithKline, 709 Swedeland Road, UW 2111, King of Prussia, Pennsylvania 19406, USA; 2 Department of Epidemiology and Biostatistics, Case Western Reserve University, Case Western Reserve University, 2103 Cornell Road, Wolstein Research Building, Room 1304, Cleveland, Ohio 44106, USA

## Abstract

A new type of test is presented for genome-wide association studies using a case-control design. It is referred to as the adaptive two-stage (ATS) analysis, being based on both the Hardy-Weinberg disequilibrium trend test (HWDTT) and the Cochran-Armitage trend test (CATT). The procedure for the ATS is to screen single-nucleotide polymorphisms (SNPs) using the HWDTT in a first stage, and then test a reduced number of SNPs that pass the screening step in a second stage using the CATT. In the Genetic Analysis Workshop 15 simulated data set, this ATS analysis captured, after Bonferroni correction, the region from 32447.149 kb to 32859.819 kb and the region around 37363.880 kb that are close to the actual trait loci on chromosome 6. We compared the ATS with other ways of combining the *p*-values of the HWDTT and the CATT, the classical form of Fisher's test and a weighted form of Fisher's test. Results showed that the proposed ATS has good performance and could detect the regions containing a susceptibility locus.

## Background

The advance of genotyping technologies and reduction of genotyping costs are resulting in genome-wide association studies using 100,000 to 500,000 (100 k–500 k) single-nucleotide polymorphisms (SNPs) across the whole genome in which tests for association are performed between each SNP and a disease in a case-control design. However, one of the big challenges of genome-wide association studies is the issue of multiple testing [1].

Zhang et al. [2] proposed an adaptive two-stage (ATS) analysis using two trend tests, the Hardy-Weinberg disequilibrium trend test (HWDTT) and the Cochran-Armitage trend test (CATT). All samples are used in both stages in the same way that Van Steen et al. [3] applied a two-stage analysis of family-based association. The adaptive two-stage analysis proposed here uses the HWDTT in the first stage to screen the SNPs and then tests a reduced number of SNPs that pass this screening step using the CATT in the second stage. The conservative Bonferroni-corrected *p*-value of an allele-based test is obtained for each SNP, but it is only necessary to correct for the number of SNPs included in the second-stage analysis.

As an alternative approach, Fisher's combination of *p*-values, referred to as Fisher's test, was considered by Zhang et al. [2]. Because the HWDTT and CATT are asymptotically independent under the null hypothesis, Fisher's test statistic [4] is given by *T *= -2log(*p*_*HWDTT*_) - 2log(*p*_*CATT*_), which under the null has a chi-square distribution with 4 df. Recently, Hwang et al. [5] extended Fisher's test to a "weighted" version that aims to maximize overall statistical power for a given significance level (0 ≤ *α *≤ 1) using a nonparametric distribution with a Gaussian Kernel density.

In this study, we applied the ATS analysis to the Genetic Analysis Workshop (GAW15) simulated data set in order to find susceptibility disease genes. We compared the results of the ATS with those of the CATT, the HWDTT, the classical Fisher test (CFT), and the weighted Fisher test (WFT). The ATS was Bonferroni-corrected for multiple testing, so, for the sake of comparison, the CATT, HWDTT, and Fisher tests (CFT and WFT) are also adjusted using the same correction method.

## Methods

For the ATS analysis of association, Zhang et al. [2] applied the HWDTT and the CATT to case-control studies. Song and Elston [6] and Zhang et al. [2] showed that these two statistics are asymptotically independent under the null hypothesis of no association. Therefore, they used all samples for both stages of the analysis. For the first stage of the proposed ATS analysis, the HWDTT is applied to test each SNP at the significance level *α*_1 _chosen on the basis of the conditional power of the HWDTT. The smallest *α*_1 _is chosen such that the power is at least 1 - *β*, where *β *is the type II error.

Denote the estimators of the genotype frequencies in cases and controls ${\hat{p}}_{i}=r_{i}/r$ and ${\hat{q}}_{i}=s_{i}/s$ for *i *= 0, 1, 2, so that ${\hat{p}}_{A}={\hat{p}}_{2}+{\hat{p}}_{1}/2$ and ${\hat{q}}_{A}={\hat{q}}_{2}+{\hat{q}}_{1}/2$ are estimators of the frequencies of the allele A in cases and controls. Song and Elston [6] considered the difference in disequilibrium coefficients between cases (*D*_1_) and controls (*D*_0_), where *D*_1 _= *p*_2 _- (*p*_2 _+ *p*_1_/2)^2 ^and *D*_0 _= *q*_2 _- (*q*_2 _+ *q*_2_/2)^2^. The HWDTT statistic can be written as

THWDTT=ZHWDTT2V^ar(Z)HWDTT=rsn3[(p^2−p^A2)−(q^2−q^A2)]2{n−(n2+n1/2)}2(n2+n1/2)2,

where *n*_*i *_= (*r*_*i *_+ *s*_*i*_) and $n=\sum_{i=0}^{2}n_{i}$. The asymptotic power of the HWDTT can then be written as

$$\pi =\Phi \left(\frac{-z_{1-\alpha_{1}/2}\sigma_{0}-\sqrt{n}(D_{1}-D_{0})}{\sigma_{H}}\right)+1-\Phi \left(\frac{z_{1-\alpha_{1}/2}\sigma_{0}-\sqrt{n}(D_{1}-D_{0})}{\sigma_{H}}\right),$$

where

*f*(*a*, *b*) = (1 - 2*b *- *a*)^2^*b*(1 - *b*) + 2*ab*(b + *a*/2)(1 - 2*b *- *a*) + (*b *+ *a*/2)^2^*a*(1 - *a*),

$\sigma_{0}^{2}$ = *f*(*r'p*_1 _+ *s'q*_1_, *r'p*_2 _+ *s'q*_2_)/(*r's'*), *r' *≈ *r*/*n *and *s' *≈ *s*/*n*,

$\sigma_{H}^{2}$ = *nVar*(*D*_1 _- *D*_0_),

Φ is the distribution function of the standard normal *N*(0, 1), and

$z_{1-\alpha_{1}/2}$ is the 100(1 - *α*_1_/2)^th ^percentile of *N*(0, 1).

The SNPs for which the null hypotheses are rejected in the first stage are tested in the second stage analysis by the CATT at the level *α*_2 _= *α'*/(*mα*_1_), where *α' *is obtained by the parametric bootstrap to control the overall type I error rate of the ATS analysis. Then *α*_2 _controls the overall type I error rate to *α *(taken to be 0.05) for a total of *m *simultaneous hypothesis tests (SNPs). As in Van Steen et al. [3], the overall *p*-value of the ATS is the *p*-value of the second analysis, which here is the CATT.

### Data

We used the GAW15 simulated Problem 3 data set for rheumatoid arthritis (RA), which includes 100 replicates. Each replicate contains 1500 families with an affected sibling pair and 2000 unaffected control subjects. To obtain a sample of cases and controls, we randomly chose one case from each affected sib pair. Thus, from each replicate we selected 1500 cases with RA and 2000 controls. In order to compare the performance of all methods in a small sample size, we randomly sampled 200 cases and 200 controls from each of the 100 replicate samples of 1500 cases and 2000 controls. To examine type I error rate, we concentrated on 100 markers that were at least 20,000 kb distant from the identified peaks on chromosome 6. Therefore, the total number of marker tests to examine type I error was 10,000 (100 markers × 100 replicates). To examine power, we concentrated on all 674 markers on chromosome 6 using only one replicate of 200 cases and 200 controls. Among these 674 markers, 5 markers are causative, in the region between 32447.149 kb and 37363.880 kb on chromosome 6.

## Results

The results for type I error are shown in Table 1 for the nominal significance levels *α *= 0.05 and *α *= 0.001. Table 1 shows that all the test statistics have nominal significance level close to the actual significance levels.

**Table 1:** Type I error rates

Test statistics	*α *= 0.05	*α *= 0.001
Hardy-Weinberg disequilibrium trend test	0.0468	0.0013
Cochran-Armitage trend test	0.0488	0.0008
Classical Fisher's test	0.0503	0.0017
Weighted Fisher's test	0.0502	0.0010
Adaptive two-stage	0.0492	0.0012

**Figure 1:**
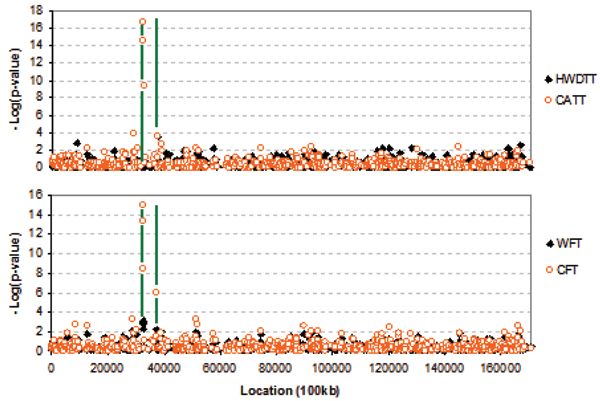
Results of SNP markers using HWDTT, CATT, CFT, and WFT for chromosome 6.

Without multiple testing corrections, the HWDTT, CATT, CFT, and WFT showed strong associations (*p *< 0.001) with a susceptibility disease gene in the region (Fig. 1, green line) between 32447.149 kb and 32859.819 kb on chromosome 6. In addition, the HWDTT, CATT, and Fisher tests (CFT and WFT) showed a significant association (*p *< 0.05) at 37363.880 kb, which was close to a trait locus (37233.784 kb). The results also indicated that i) CATT and CFT have similar power near the peak, ii) CFT is more powerful than WFT near the peak, and iii) the HWDTT is not powerful near the peak.

With Bonferroni multiple testing correction, Table 2 shows the locations of the SNP markers that are significantly associated with RA. In Table 2, the *p*-values for HWDTT, CATT, WFT, and CFT are compared at the significance level 0.05/674 = 7.42 × 10^-5^. To obtain the *p*-value of the ATS analysis, first we calculated the asymptotic power of the HWDTT for *α*_1 _= 0.01 to 1.00 with increments of 0.01 for each of the 674 SNPs. Then for each SNP we chose the *α*_1 _that had at least 80% conditional power. Results showed that 535 SNPs had *p*-values of HWDTT less than this *α*_1 _and these were analyzed in the second stage. Then we obtained the adjusted overall level for *α' *using the parametric bootstrap with increments of 0.001 and 10,000 replications. For example, for 80% conditional power, the SNP in location 32447.149 kb requires *α*_1 _= 0.77 in the first stage. Then, based on the bootstrap, we obtained *α' *= 0.03. Thus, the adjusted level required for the second stage is *α*_2 _= 0.03/(674 × 0.77) = 5.78 × 10^-5^. The *p*-values of the HWDTT is 0.53 <*α*_1 _= 0.77. Thus, the SNP in location 32447.149 kb is significant in the first stage. The *p*-value of the CATT in the second stage is 2.7 × 10^-15 ^<*α*_2 _= 5.8 × 10^-5^. Hence, the SNP in location 32447.149 kb is significant when the adaptive two-stage analysis is applied.

**Table 2:** The locations of the SNP markers that are significantly associated with RA after Bonferroni correction at the significance level *α *= 0.05

					ATS^b^	
					
Location (kb)	HWDTT^a^	CATT^a^	WFT^a^	CFT^a^	Stage 1^c^	Stage 2^d^
32447.149	NS^e^	2.65 × 10^-15^	NS	4.96 × 10^-14^	0.53 < 0.77	2.7 × 10^-15 ^< 5.8 × 10^-5^
32499.465	NS	<1.0 × 10^-17^	NS	<1.10 × 10^-16^	0.65 < 0.83	1.0 × 10^-17 ^< 6.3 × 10^-15^
32521.277	NS	<1.0 × 10^-17^	NS	<1.10 × 10^-16^	NS	NS
32772.203	NS	5.36 × 10^-15^	NS	3.44 × 10^-9^	0.27 < 0.65	5.4 × 10^-15 ^< 5.7 × 10^-5^
37363.880	NS	NS	NS	1.19 × 10^-6^	0.0003 < 0.02	0.0003 < 0.0011

^a^The *p*-values should be compared with 0.05/674 = 7.42 × 10^-5^.

^b^The *p*-value should be compared with the optimal level in the two stages.

^c^The level in the first stage with conditional power at least 80% (*α*_1_)

^d^The adjusted level for the second stage (*α*_2 _= *α'*/(674*α*_1_))

^e^NS: no significance

Applying the ATS with the optimal *α*_2_, the three SNPs in the region between 32447.149 kb and 32772.203 kb were associated with RA. In particular, after Bonferroni correction the ATS showed that the SNP at 37363.880 kb was associated with RA. In the region between 32447.149 kb and 32859.819 kb, the CATT and CFT had Bonferroni-corrected average *p*-values < 1.34 × 10^-12 ^and *p *< 5.80 × 10^-7^, respectively. In addition, the corrected *p*-value of CFT is 0.000802 for a marker at location 37363.880 kb. However, the Bonferroni-corrected *p*-value of WFT is not significant. This could indicate that WFT with Bonferroni correction may be too conservative, because it already minimizes the number of false positives and false negatives.

## Discussion

We have presented the use of a new method for genome-wide association studies with optimal choice of significance level to maximize the power and at the same time asymptotically control the overall type I error. The ATS analysis uses two independent test statistics-here the HWDTT and the CATT. We compared the performance of the HWDTT, CATT, Fisher's tests combining the HWDTT and CATT, and the ATS in this data set with Bonferroni correction for multiple testing and found that the ATS had good performance. Compared to WFT or CFT, the ATS showed higher power in this study. CFT captured the region that is close to a trait locus and gave higher power than WFT, but had *p *< 0.05 in regions that were distant from the true locations, so that the falsepositive rate of CFT seems to be higher than that of WFT in these data. This agrees with the original paper [5] reporting a lower false-positive rate for WFT.

In this study, we applied all test statistics to the GAW15 simulated data. However, the simulated effect near the peak is so strong that all test statistics were able to detect the susceptibility disease gene on chromosome 6 with a sample size as small as 200 cases and 200 controls; the Bonferroni corrected *p*-values were significant for the CATT, CFT, and ATS. Zhang et al. [2] showed that the ATS is more powerful than the CATT and CFT when applied to real data in an association study of 96 cases and 50 controls that used 103,611 SNPs for a genome-wide association study of age-related macular degeneration [7].

Finally, we should note the limitations of the ATS method we used. The ATS analysis will be more costly than other two-stage analysis studies, which uses separate portions of the sample for each stage, because the ATS analysis uses all subjects in both stages. In addition, the ATS analysis is more computationally intensive than the other tests because it is a necessary to obtain the adjusted overall level for *α' *using the parametric bootstrap.

## Conclusion

Using the ATS analysis, a sample size as small as 200 cases and 200 controls showed good performance after Bonferroni correction for association with a susceptibility disease gene in the region between 32447.149 kb and 37363.880 kb on chromosome 6.

## Competing interests

The author(s) declare that they have no competing interests.
